# The effects of stretch activation on ionic selectivity of the TREK-2 K2P K^+^ channel

**DOI:** 10.1080/19336950.2017.1356955

**Published:** 2017-07-19

**Authors:** Ehsan Nematian-Ardestani, Viwan Jarerattanachat, Prafulla Aryal, Mark S. P. Sansom, Stephen J. Tucker

**Affiliations:** Clarendon Laboratory, Department of Physics and OXION Initiative in Ion Channels and Disease, University of Oxford, Oxford, UK

**Keywords:** K2P channel, K^+^ channel, KCNK10, TREK-2, Ion selectivity, Mechanosensitive

## Abstract

The TREK-2 (*KCNK10*) K2P potassium channel can be regulated by variety of polymodal stimuli including pressure. In a recent study, we demonstrated that this mechanosensitive K^+^ channel responds to changes in membrane tension by undergoing a major structural change from its ‘down’ state to the more expanded ‘up’ state conformation. These changes are mostly restricted to the lower part of the protein within the bilayer, but are allosterically coupled to the primary gating mechanism located within the selectivity filter. However, any such structural changes within the filter also have the potential to alter ionic selectivity and there are reports that some K2Ps, including TREK channels, exhibit a dynamic ionic selectivity. In this addendum to our previous study we have therefore examined whether the selectivity of TREK-2 is altered by stretch activation. Our results reveal that the filter remains stable and highly selective for K^+^ over Na^+^ during stretch activation, and that permeability to a range of other cations (Rb^+^, Cs^+^ and NH_4_^+^) also does not change. The asymmetric structural changes that occur during stretch activation therefore allow the channel to respond to changes in membrane tension without a loss of K^+^ selectivity.

## Introduction

Two-Pore Domain (K2P) potassium channels are often described as a simple background “leak” conductance. However, several members of this family exhibit “polymodal” regulation by a diverse range of physical and chemical stimuli such as pressure, temperature, voltage, pH, lipids, anesthetics and various second messengers.^1,2^ Such diverse stimuli allow these channels to integrate a wide variety of signaling pathways into changes of electrical signaling. For example, activation of the mechanosensitive K2P channels (TREK-1, TREK-2 and TRAAK) by membrane stretch allows them to couple mechanical forces to changes in membrane potential. This is particularly important in the heart where the TREK-1 and TREK-2 channels are thought to contribute to the stretch-activated potassium currents involved in mechano-electrical coupling and arrythmogenesis.^3,4^ Their mechanosensitivity may also be important in some populations of sensory neurons for the sensation of pain^5^ and a mutation within the filter of TREK-1 associated with ventricular tachycardia has also been reported to result in increased Na^+^ permeability and a hypersensitivity to stretch activation.^6^ As a result, there is considerable interest in understanding the molecular mechanisms that underlie K2P channel gating by membrane stretch.

Fortunately, crystal structures now exist for several different K2P channels, including TREK-2 and TRAAK,^7-10^ thereby allowing several recent studies to directly address the molecular mechanisms underlying these processes.^11-13^ In one of these studies, we used a series of molecular dynamics simulations to demonstrate that activation of the TREK-2 channel by membrane stretch involves movement from the “down” conformation to a more expanded “up” conformation within the bilayer.^11^ Similar changes also appear to underlie stretch-activation of the TRAAK channel.^12^ Interestingly, although membrane stretch increases tension in both leaflets of the bilayer, these structural changes occur primarily within the lower half of the protein, presumably to prevent distortion of the K^+^ selectivity filter during mechanical activation.

Unlike classical K^+^ channels, K2P channels appear to lack a lower bundle-crossing gate and their primary gating mechanism is located within or near the selectivity filter.^14-17^ Consequently, regulation of this filter gate is thought to be coupled to movement of the transmembrane helices during mechanogating.^14,15,18,19^ The precise mechanisms are unknown, but must involve some form of structural change, or change in conformational stability, within the filter itself. As a result, such changes not only have the potential to alter gating within the filter, but also ionic selectivity. Interestingly, several K2P channels have been reported to exhibit dynamic changes in their ionic selectivity during channel gating,^20^ this includes the TREK channels which have been proposed to exhibit permeability to Na^+^ and even glutamate ions under certain conditions.^21,22^ In this addendum to our original study we have therefore examined the structural changes that occur within the selectivity filter upon stretch activation, and have measured how this influences the ionic selectivity of TREK-2.

## Results and discussion

### Structural consequences of stretch activation

**Figure 1.**
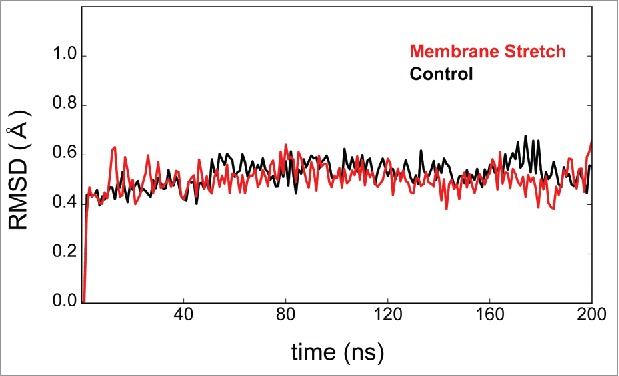
Relative stability of the selectivity filter during stretch activation. Examples of the structural changes occurring during simulation of the TREK-2 down state structure in the presence (red) or control (black) in the absence of applied membrane stretch (P-50 protocol). The Root Mean Squared Deviation (RMSD) was calculated by comparison to the starting structure (PDB ID: 4XDJ) and refers to changes in the position of the Cα atoms within both the canonical K^+^ filter sequences for the 2 pore domains (P1: TIGYGN; P2: TVGFGD). The sequences appear equally stable in the presence and absence of membrane stretch. The filter therefore appears relatively isolated from the larger-scale structural changes that occur elsewhere in the channel.

In our previous study, we used a series of molecular dynamics simulations to examine the effects of membrane stretch on the structure of TREK-2^11^). We observed that although the stretch-induced conformational changes were mostly restricted to the lower half of the TMs (i.e. that part of the protein within the inner leaflet of the bilayer) they were still capable of producing a change in the distribution of K^+^ ions within the selectivity filter. This obviously has the potential to alter ionic selectivity as well as gating, and to evaluate these structural changes in more detail we calculated the root-mean-square deviation (RMSD) of the Cα atoms within the canonical K^+^ filter sequences for the 2 pore domains (P1: TIGYGN; P2: TVGFGD) during these simulations. Comparison against the starting down state structure revealed that when stretched the average RMSD was 0.586 ± 0.265 Å over the course of 200 ns in the 4 separate simulations that were performed. However, this was not significantly different to the values calculated in the absence of membrane stretch (0.517 ± 0.112 Å) and examples are shown in Fig. 1. These changes are relatively small compared with the stretch-induced movements seen elsewhere in the transmembrane helices, but still have the potential to alter ionic selectivity and so we next examined whether this changes during stretch activation.

### Ionic selectivity of stretch activated TREK-2

To assess possible changes in ionic selectivity we conducted a series of biionic measurements for a range of monovalent cations with different permeability. Inside-out patches from *Xenopus laevis* oocytes expressing human TREK-2 channels were measured using voltage ramps from -80 mV to +120 mV to measure the full range of possible reversal potentials for these different ions, as well as any shifts that may occur during stretch activation. Control measurements in the absence of applied pressure were taken immediately before application of negative pressure (-22 mmHg, applied via the patch pipette). This pressure produces near maximal activation of channel activity. The pipette solution contained 120 mM K^+^ pH 7.4, while the bath solution contained equal concentrations (120 mM) of either K^+^, Na^+^, Rb^+^, Cs^+^, or NH_4_^+^ (pH 8.0) as the intracellular ion. An intracellular pH of 8.0 was chosen to avoid possible interference by pH activation. Under these conditions TREK channels are mostly closed at voltages below the reversal potential.^16^ Consequently, any changes in the reversal potential should therefore reflect changes in ion selectivity within the filter during stretch activation.

**Figure 2.**
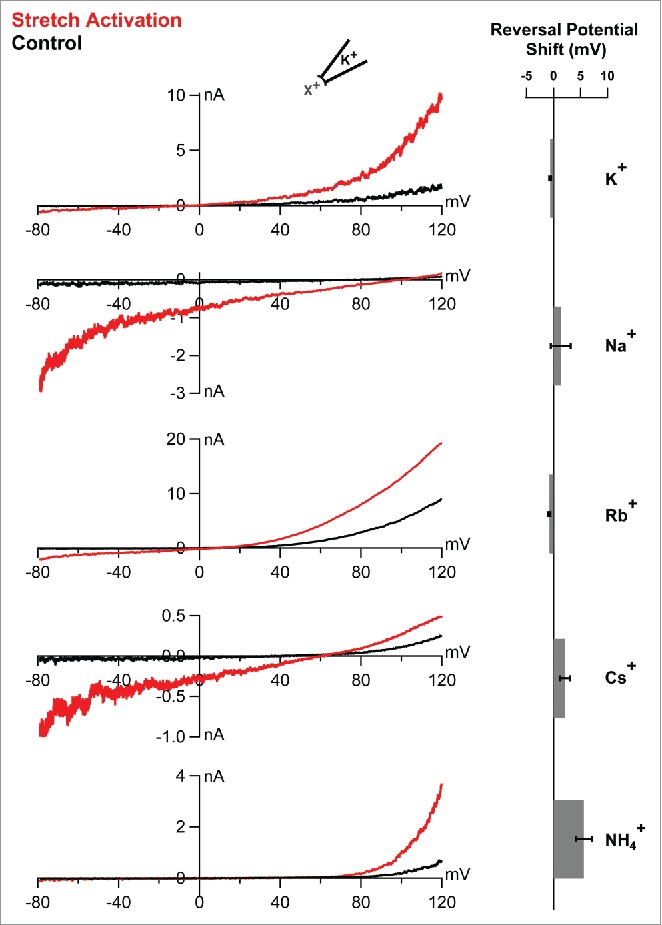
Stretch activation does not alter the ionic selectivity of TREK-2. Left panel shows macroscopic currents recorded in a single representative giant patch excised from oocytes expressing the human TREK-2 channel under biionic conditions. A voltage ramp from -80 mV to +120 mV was used to measure the full range of possible reversal potential for the different ions indicated. Currents were recorded in either the presence (red) or absence (black) of membrane stretch. Stretch was induced by application of negative pressure applied via the patch pipette. The right panel shows the shifts in reversal potential as calculated and plotted for each of the respective ions. Error bars represent the mean ± SEM. None of these shifts are statistically significant.

Figure 2 shows macroscopic currents recorded before and after stretch activation of TREK-2 in the presence of these different intracellular ions. As we have described previously, when Rb^+^ is used as the intracellular ion, large outward currents are observed at depolarized potentials in the absence of membrane stretch. These Rb^+^ currents are larger than those seen with K^+^ as the permeant ion due to activation of the filter gating mechanism^16^ and could also be activated in response to membrane stretch. In our most recent study, we reported a positive synergy between activation by Rb^+^ and membrane stretch.^11^ Interestingly, this effect shares many similarities with the synergy seen between voltage and stretch activation of the related TRAAK channel.^23^ However, examination of the reversal potential upon stretch activation with Rb^+^ as the permeant ion revealed no shift (Fig. 2).

When Na^+^ was used as the intracellular ion, we also observed no significant change in reversal potential upon stretch-activation, and the channels remained essentially impermeant to Na^+^. Likewise, when Cs^+^ was used no shift was observed (Fig. 2). Even the limited permeability of NH_4_^+^ was unaffected by stretch activation and the reversal potential shifted only ∼6mV more positive correlating with a possible increase, not decrease in K^+^ selectivity. However, this shift was not significant (p<0.0005) and likely results from the fact that the ramp currents run very close to zero current level up to the vicinity of the reversal potential. These measurements therefore demonstrate that levels of stretch activation that produce near maximal activation in giant excised patches do not change the ionic selectivity of TREK-2. Furthermore, near-lytic tensions within the bilayer do not appear to distort the structure of the filter. This structural and functional stability will clearly be important for many of the proposed physiologic functions of these mechanosensitive K2P channels. This includes their role as stretch-activated potassium currents in the heart where any changes in ionic selectivity would compromise their ability to influence the shape and duration of the action potential,^3,4,24^ and where sodium leak may contribute to cardiac arrhythmia.^25^

Overall, the results reveal that the structural and functional integrity of the K^+^ selectivity filter is maintained during mechanogating. Thus despite previous reports of dynamic selectivity in several different types of K2P channels, including Na^+^ and even glutamate permeability in TREK-1, we find no evidence for any changes in the ionic selectivity of TREK-2 during mechanogating. These results are also consistent with previous reports that TREK channels remain highly selective for K^+^ when activated by BL1249.^26^ Thus, even though activation of the filter gate must require some degree of movement or change in conformational stability of the filter itself, such changes do not appear sufficient to alter the basic function of this structural motif, i.e., its ability to confer a high degree of selectivity for K^+^ ions.

## Methods

### Computational methods

The molecular dynamics simulations analyzed in this study were performed as described in our previous study (11). Briefly, this involved all-atom simulations of the down state of the human TREK-2 channel (PDB ID: 4XDJ) embedded in a POPC bilayer in response to the P-50 membrane stretch protocol. The RMSD analysis involved comparison of Cα-atoms of pore-loop residues 172–177 and 281–286 before and after membrane stretch.

### Electrophysiology

All methods used for the measurement of TREK-2 channels expressed in *Xenopus* oocytes have been described previously.^11,16^ Briefly, pipettes with a resistance of 0.3–0.9 MΩ (tip diameter of 5–15 µm) were filled with a standard pipette solution (in mM): 120 KCl, 10 HEPES and 3.6 CaCl_2_ (pH 7.4 adjusted with KOH/HCl). The intracellular (bath) solution had the following composition in (mM): 120 KCl, 10 HEPES and 2 EDTA (pH 8.0 adjusted with KOH/HCl). In other intracellular solutions the K^+^ was replaced by Na^+^, NH_4_^+^, Cs^+^ or Rb^+^ and the pH adjusted with hydroxide of the relevant ion species. Currents were measured using voltage ramps from -80 mV to +120 mV.
